# Ginsenoside Rh2 Mitigates Endoplasmic Reticulum Stress-Induced Apoptosis and Inflammation and Through Inhibition of Hepatocyte–Macrophage Inflammatory Crosstalk

**DOI:** 10.3390/nu17101682

**Published:** 2025-05-15

**Authors:** Shinjung Park, Inae Jeong, Ok-Kyung Kim

**Affiliations:** 1 Division of Food and Nutrition, Chonnam National University, Gwangju 61186, Republic of Korea; qkrtlswjd1@jnu.ac.kr (S.P.); wjddlsdo2@jnu.ac.kr (I.J.); 2 Human Ecology Research Institute, Chonnam National University, Gwangju 61186, Republic of Korea

**Keywords:** endoplasmic reticulum stress, ginsenoside Rh2, NAFLD, macrophage

## Abstract

**Background/Objectives:** Endoplasmic reticulum stress (ERS) contributes to hepatocyte inflammation, triggered by prolonged exposure to lipotoxicity, and promotes non-alcoholic fatty liver disease (NAFLD) progression by recruiting and activating hepatic macrophages, which accelerate fibrosis and exacerbate disease progression. Here, we aimed to evaluate the therapeutic potential of ginsenoside Rh2 (Rh2) in a cell model of NAFLD induced by the ERS inducer thapsigargin (THA). **Methods:** HepG2 cells were treated with THA to induce ERS and mimic NAFLD conditions. The effects of Rh2 on ERS, lipid accumulation, and apoptosis were assessed in HepG2 cells. Additionally, THP-1 cells were used to investigate macrophage activation upon exposure to conditioned medium (CM) from THA- and Rh2-treated HepG2 cells. Gene and protein expression of inflammatory and lipid synthesis markers were analyzed, as well as M1/M2 macrophage polarization markers. **Results:** Rh2 inhibited THA-induced apoptosis, ERS, and lipid accumulation in HepG2 cells. It also reduced the expression of lipid synthesis genes (SREBF1, FAS) and inflammatory markers (IL-6, IL-1β, TNF-α, MCP-1). CM from Rh2-treated HepG2 cells suppressed macrophage activation in THP-1 cells, decreased M1 polarization markers (CD80, CD86), and increased M2 markers (CD163, Arg1, MRC-1). **Conclusions:** These results suggest that Rh2 effectively suppresses inflammation and lipid storage in ERS-induced HepG2 cells while modulating the crosstalk between hepatocytes and macrophages. These findings underscore the potential of Rh2 as a promising therapeutic agent for the prevention and early intervention of NAFLD progression.

## 1. Introduction

The rapid changes in dietary habits and lifestyles have contributed to the rapid increase in metabolic disorders worldwide. Among them, nonalcoholic fatty liver disease (NAFLD) has emerged as a major public health problem, with a global prevalence of approximately 30% [1], and approximately 20% of them progress to nonalcoholic steatohepatitis (NASH) [2,3]. Endoplasmic reticulum stress (ERS), in particular, is frequently observed in the livers of patients with NAFLD [4], and is considered a key contributor to the progression from steatosis to NASH. Experimental evidence demonstrates that the treatment of hepatocytes with the ERS inducer thapsigargin (THA) upregulates the expression of key regulators such as XBP1 and FOXA3, along with genes involved in lipid synthesis [5]. Moreover, ERS activates lipid synthesis pathways [6,7,8,9,10] including that of SREBP1c/ACC/FAS, leading to triglyceride accumulation and the subsequent activation of inflammatory pathways such as JNK and NF-κB [11,12,13]. These processes collectively establish a chronic inflammatory environment, further amplifying hepatocellular damage.

Emerging evidence highlights the critical role of hepatocyte–macrophage crosstalk in the progression of NASH. In particular, inflammation induced by fatty liver exacerbates liver damage and fibrosis by secreting various inflammatory cytokines and promoting macrophage recruitment and infiltration into liver tissue [14,15,16,17]. These activated macrophages differentiate into classical macrophages (also known as M1 macrophages) or alternative macrophages (also known as M2 macrophages). In the early stage of NAFLD, M1 macrophages stimulate hepatic stellate cells (HSCs), contributing to fibrosis [18,19,20,21]. These pathological interactions accelerate NAFLD progression and increase the risk of liver-related morbidity and mortality [22]. Given these findings, therapeutic strategies aimed at attenuating ERS, reducing inflammation, and inhibiting macrophage activation and infiltration are considered crucial for effectively managing and preventing NAFLD progression.

In recent years, natural plant chemical extracts have been identified as promising alternatives for alleviating the side effects and complications of existing disease-treating drugs [23]. Ginseng, a root plant cultivated in several countries in East Asia and North America, has been used for thousands of years in traditional Asian medicine to prevent and treat various physiological and pathological conditions. Notably, the level of ginsenoside Rh2 (Rh2), which is present only in trace amounts or is undetectable in unprocessed ginseng, is markedly increased in red ginseng. Rh2 has been reported to provide various health benefits, including anticancer [24,25], antidiabetic, anti-Alzheimer’s disease [26], and anti-inflammation effects [27,28]. However, research on the role of Rh2 in the progression of NAFLD, particularly in relation to macrophage recruitment and polarization, remains limited. Further mechanistic investigations are warranted to elucidate its therapeutic potential in this context. Recent studies have shown that Rh2 effectively reduces ERS-related proteins and alleviates ERS induced by ERS inducers. Additionally, Rh2 has been shown to attenuate adipocyte differentiation in both rodent and human preadipocyte cell lines, and dietary Rh2 intake has been observed to inhibit adipogenesis in obese mice [29,30]. Therefore, this study aimed to investigate whether Rh2, recognized for its anti-inflammatory properties [31,32], could regulate lipogenesis and mitigate inflammation in ERS-induced HepG2 cells. Furthermore, it examined whether Rh2-treated hepatocytes could modulate hepatic macrophage infiltration and activation, thereby alleviating cellular damage and preventing the progression of NAFLD.

## 2. Materials and Methods

### 2.1. Reagents

Rh2 (purity ≥ 97%), Compound K (purity ≥ 96%), Panaxadiol (PD) (purity ≥ 96%), Palmitic acid (PA), and Ethanol (EtOH) were purchased from Sigma-Aldrich (St. Louis, MO, USA). Protopanaxadiol (PPD) (purity ≥ 90%) was purchased from PytoLab (Vestenbergsgreuth, Germany). Thapsigargin (THA) was purchased from Thermo Fisher Scientific (Waltham, MA, USA).

### 2.2. Cell Culture and Stimulation

HepG2 cells and THP-1 cells were purchased from ATCC. HepG2 cells were cultured in Dulbecco’s modified Eagle’s medium (DMEM; Gibco, Waltham, MA, USA) supplemented with 10% fetal bovine serum (FBS; Gibco) and 1% penicillin/streptomycin (Gibco). THP-1 cells were cultured in Roswell Park Memorial Institute 1640 medium (RPMI 1640; Gibco) supplemented with 10% heat-inactivated FBS (Gibco) and 1% penicillin/streptomycin (Gibco). The cells were always washed with FBS before experiments to reduce interference from unknown substances in the serum and to ensure a homogeneous cell population. All cells were cultured in a humidified 5% CO_2_ environment at 37 °C. To induce stress in HepG2 cells, the cells were treated with 60 μM PA, 1000 μM EtOH, and 2 μM THA. After incubation for 42 h or 48 h, the cells were harvested and used for experiments. Cells at passages 12–16 were used for all experiments.

### 2.3. Cell Viability Assay

HepG2 cells were seeded in 96-well plates (Falcon) at a density of 3 × 10^4^ cells per well. For the ginsenoside compound cell viability assay, HepG2 cells were treated with both 2 μM THA and Rh2 (2.5–10 μM), Compound K (0–3 μM), PD (0–15 μM), and PPD (0–15 μM) for 48 h. After washing the cells, 200 μL of fresh medium was added per well. Cell viability was assessed using a water-soluble tetrazolium salt-based EZ-Cytox assay kit (Dogenbio, Seoul, Republic of Korea) according to the manufacturer’s instructions.

### 2.4. Apoptosis Assay

Apoptotic cells were detected using Annexin V (FIFC; BD bio, San Jose, CA, USA) and propidium iodide (PI; BD bio). HepG2 cells were stained with 5 μL of Annexin V and PI for 15 min at RT while protected from light. Stained cells were analyzed with the CytoFLEX flow cytometer (Bexkman Coulter, Brea, CA, USA) within 1 h.

### 2.5. Oil Red O Staining

ERS-induced lipid accumulation in HepG2 cells was visually confirmed by staining lipid droplets using Oil Red O solution (Sigma-Aldrich, St. Louis, MO, USA). The Oil Red O working solution was diluted with distilled water at a 4:6 ratio and filtered through a 0.2 μM filter for 20 min, followed by washing twice with DW. The cells were incubated with 60% isopropanol for 5 min, removed by suction, and completely left alone for 1 h in the dark. After washing 4 times with DW, the stained lipid droplets were photographed using a microscope. The stained lipid droplets were extracted by adding 100% isopropanol and quantified by measuring the absorbance at 510 nm. The values were corrected based on cell viability. All experiments were performed at room temperature.

### 2.6. Triglycerides Assay

HepG2 cells were harvested after washing with PBS. To quantify intracellular triglycerides, 5% NP-40 was added to the harvested cells. The mixture was heated at 80–100 °C for 5 min, cooled, and then heated again for 5 min. Then, the mixture was centrifuged at 3000× *g* for 20 min at 4 °C. Subsequently, the supernatant was separated, diluted 10 times in DW, and used for experiments. Intracellular triglyceride accumulation was quantified according to the protocol of the PicoSens™ Triglyceride Assay Kit (Biomax, Guri, Republic of Korea). The values were corrected based on cell viability.

### 2.7. Protein Extraction and Immunoblotting

Protein extraction was performed using RIPA lysis buffer (Rockland, Pottstown, PA, USA) containing protease and phosphatase inhibitors (Thermo Fisher Scientific, Waltham, MA, USA). After centrifugation at 15,000× *g* for 20 min at 4 °C, the resulting supernatant was collected. Protein quantification was performed by Bradford protein assay (Bio-Rad, Hercules, CA, USA), and equal amounts of protein were subjected to SDS-PAGE. The proteins were subsequently transferred to polyvinylidene difluoride membranes (Bio-Rad). The membranes were blocked with 5% skim milk in Tris-buffered saline with 0.1% Tween^®^ 20 detergent (TBST) at room temperature for 1 h, followed by overnight incubation with primary antibodies against XBP1s (Cell signaling, Beverly, MA, USA), FAS (Cell signaling), and β-actin (Cell signaling) at 4 °C. Then, the membranes were incubated with horseradish peroxidase-linked secondary antibodies (Bio-Rad) at RT for 1 h. Enhanced chemiluminescence (Bio-Rad) was employed for blot visualization using the ChemiDoc Imaging System (Bio-Rad). The resulting bands were quantified using National Institutes of Health Image J software (v1.53e).

### 2.8. Harvest of Conditioned Medium (CM) from HepG2

HepG2 cells were stimulated with 2 μM THA and either 2.5 or 5 μM Rh2 for 48 h. After washing with PBS, fresh culture medium was added and incubated for 24 h to collect the medium. Then, the CM was obtained after centrifugation (200× *g*, 10 min, 20 °C).

### 2.9. Enzyme-Linked Immunosorbent Assay (ELISA)

The concentrations of IL-6, TNF-α, IL-1β, and MCP-1 in the CM obtained from HepG2 cells were determined using ELISA kits (R&D Systems, Minneapolis, MN, USA; DY206, DY210, DY201, and DCP00). CM and standards were dispensed into 96-well plates pre-coated with capture antibodies. The levels of each cytokine and chemokine were then measured following the standard sandwich ELISA protocol provided by the manufacturer.

### 2.10. CM from HepG2 Cells Treatment in THP-1 Cells

Undifferentiated THP-1 cells were cultured at a density of 1 × 10^5^ cells in RPMI 1640 medium supplemented with 10% heat-inactivated FBS and 1% penicillin/streptomycin, mixed with conditioned medium (CM) at a 1:1 ratio. A maximum of 1 mL of CM was collected from each sample for use in the mixture, and FBS was present throughout the incubation. The medium was replenished every 48 h. On the third day of culture, the number of attached cells was counted using a microscope. Cells were harvested after 48 or 96 h of culture.

### 2.11. RNA Extraction and Reverse Transcription-Polymerase Chain Reaction (RT-PCR)

RNA extraction was performed using the RNeasy mini kit (Qiagen, Hilden, Germany). RNA quantification was conducted using NanoDrop (Quawell Technology, Palo Alto, CA, USA), and complementary DNA (cDNA) was synthesized from 100 ng or 50 ng of purified RNA using the iScript cDNA synthesis kit (Bio-rad). The reaction volume was adjusted to 20 μL following the protocol for iQ™SYBR^®^ Green Supermix (Bio-Rad). Each sample was subjected to PCR amplification using an equal amount of the cDNA template with the following steps: initial denaturation (95 °C, 3 min), followed by 40 cycles of denaturation (95 °C, 10 s) and annealing/extension (58 °C, 30 s). The CFX96 Touch Real Time-PCR Detection System (Bio-Rad) was utilized. RNA expression was normalized to β-actin expression, and relative RNA expression was calculated using the 2-ΔΔCT method. The custom-designed primers are presented in Table 1.

### 2.12. Statistical Analysis

The results are expressed as the mean ± standard deviation (SD). Statistical analysis for two-sample comparisons was performed by Student’s t-test with the 95% confidence interval (SPSS PASW Statistic 23.0; SPSS Inc., Chicago, IL, USA). Differences were considered statistically significant at *p* < 0.05.

## 3. Results

### 3.1. Inhibitory Effect of Rh2 Treatment on Apoptosis and Lipid Accumulation in ERS-Induced HepG2 Cells

**Figure 1:**
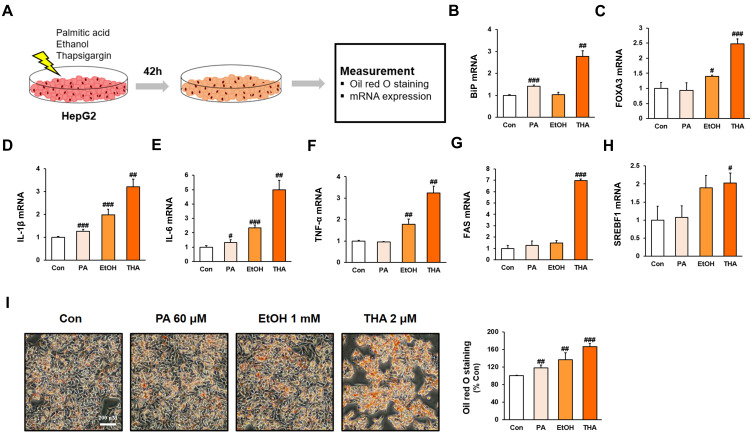
Inflammation responses and lipogenesis in HepG2 cells following treatment with PA, EtOH, and THA. HepG2 cells treated with PA, EtOH, and THA. The Con group was treated with vehicle (PBS). The identified non-cytotoxic concentrations (60 μM PA, 1000 μM EtOH, and 2 μM THA) were used in subsequent experiments. (**A**) The experiment was conducted after treating HepG2 cells with PA, EtOH, and THA, followed by incubation for 42 h. (**B**,**C**) mRNA expression of ERS markers BiP and FOXA3. (**D**–**F**) mRNA expression of inflammation markers IL-1β, IL-6, and TNF-α. (**G**,**H**) mRNA expression of FAS and SREBF1. (**I**) Comparison of lipid accumulation in HepG2 cells based on Oil Red O staining. Values are shown as mean ± SD (*n* = 3). Con vs. PA, Con vs. EtOH, Con vs. THA, # *p* < 0.05, ## *p* < 0.01, ### *p* < 0.001.

**Figure 2:**
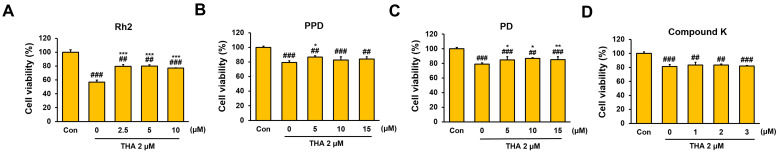
Comparison of the cytoprotective effects of ginsenosides against THA-induced ERS in HepG2 cells. Cell viability of HepG2 cells treated with 2 μM THA and ginsenosides Rh2 (0–10 μM; **A**), PPD (0–15 μM; **B**), PD (0–15 μM; **C**), and Compound K (0–3 μM; **D**) for 48h. The Con group was treated with vehicle (PBS). Values are shown as mean ± SD (*n* = 3). Con vs. 0, Con vs. 1, Con vs. 2, Con vs. 2.5, Con vs. 3, Con vs. 5, Con vs. 10, Con vs. 15, ## *p* < 0.01, ### *p* < 0.001. 0 vs. 1, 0 vs. 2, 0 vs. 2.5, 0 vs. 3, 0 vs. 5, 0 vs. 10, 0 vs. 15, * *p* < 0.05, ** *p* < 0.01, *** *p* < 0.001.

HepG2 cells were exposed to various stress inducers, including PA, EtOH, and THA, for 42 h, and then Oil Red O staining and RT-PCR analysis were performed (Figure 1A). The results demonstrated elevated expression of ER stress markers (BiP and FOXA3) and inflammatory markers (IL-6, TNF-α, and IL-1β) (Figure 1B–F). Moreover, the expression of lipogenic genes and triglyceride accumulation were significantly higher in the THA-treated group compared to other conditions (Figure 1G–I). This confirms that THA-induced ER stress is an important factor in promoting hepatic lipogenesis. Additionally, the therapeutic effects of ginsenoside compounds, including Rh2, Compound K, PD, and PPD, were evaluated. Among these compounds, Rh2 showed the most significant restoration of cell viability reduced by ER stress (Figure 2A–D, *p* < 0.001).

**Figure 3:**
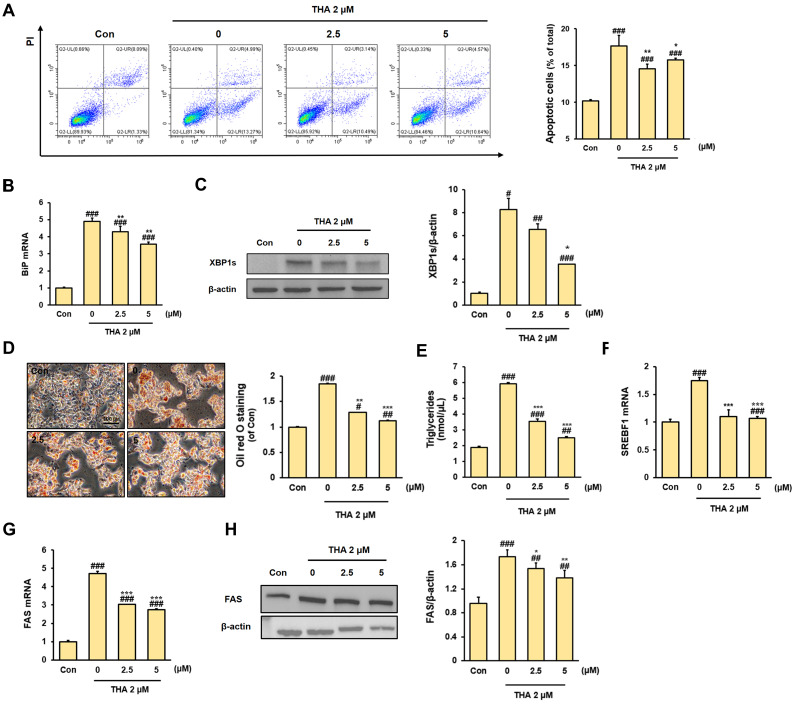
Effect of Rh2 treatment on lipogenesis and apoptosis in ERS-induced HepG2 cells. (**A**) Apoptosis of HepG2 cells treated with 2 μM THA, followed by treatment with (0 μM, 2.5 μM, 5 μM) Rh2 for 48 h. The apoptosis rate was measured by Annexin V/PI staining. mRNA expression of ERS marker (**B**) BiP, (**C**) XBP1s protein expression in HepG2 cells. (**D**) Comparison of lipid accumulation in HepG2 cells based on Oil Red O staining. (**E**) Measurement of triglycerides in HepG2 cells. mRNA expression of (**F**) SREBF1 and (**G**) FAS and (**H**) protein expression of FAS in HepG2 cells. The Con group was treated with DMSO, and the x-axis of the graph represents the Rh2 concentration. Values are shown as mean ± SD (*n* = 3). Con vs. 0, Con vs. 2.5, Con vs. 5, # *p* < 0.05, ## *p* < 0.01, ### *p* < 0.001. 0 vs. 2.5, 0 vs. 5, * *p* < 0.05, ** *p* < 0.01, *** *p* < 0.001.

Rh2 was selected for further investigation because of its excellent cytoprotective effect. Annexin V/PI staining further confirmed that Rh2 at concentrations of 2.5 and 5 μM effectively reduced cell apoptosis (Figure 3A). Next, we examined the ERS-related factors BiP and XBP1s in HepG2 cells treated with THA and exposed to Rh2. BiP was markedly increased in HepG2 cells treated with THA (0 group) compared with the Con group. However, Rh2 treatment effectively reduced BiP mRNA expression (Figure 3B). Additionally, the protein level of XBP1s was increased in the 0 group compared with the Con group, and it was reduced by Rh2 treatment (Figure 3C). The findings indicated that Rh2 treatment reduced ERS-induced cell damage.

As ERS could induce lipid accumulation in hepatocytes, we investigated whether Rh2 could exert protective effects. As expected, stronger Oil Red O staining was observed in the ERS-induced 0 group compared with the Con group. However, compared with the 0 group, the Rh2-treated group showed a weaker stained area. The results were confirmed by the quantitative measurement of Oil Red O (Figure 3D), and the triglyceride assay further supported the findings (Figure 3E). We investigated whether these effects were caused by changes in the lipid synthesis pathway. The expression level of SREBF1 mRNA in the 0 group, which was increased more than two-fold compared with the level of the Con group, was decreased following Rh2 treatment (Figure 3F). Additionally, the expression level of FAS mRNA in the 0 group, which was increased approximately five-fold compared with the level of the Con group, was significantly decreased with Rh2 treatment (Figure 3G, *p* < 0.001). Similar results were observed for the FAS protein level (Figure 3H). Overall, the results demonstrated that Rh2 treatment suppressed upregulated lipid synthesis induced by ERS, thereby preventing excessive lipid accumulation.

### 3.2. Inhibitory Effect of Rh2 Treatment on Inflammation and Fibrosis in ERS-Induced HepG2 Cells

**Figure 4:**
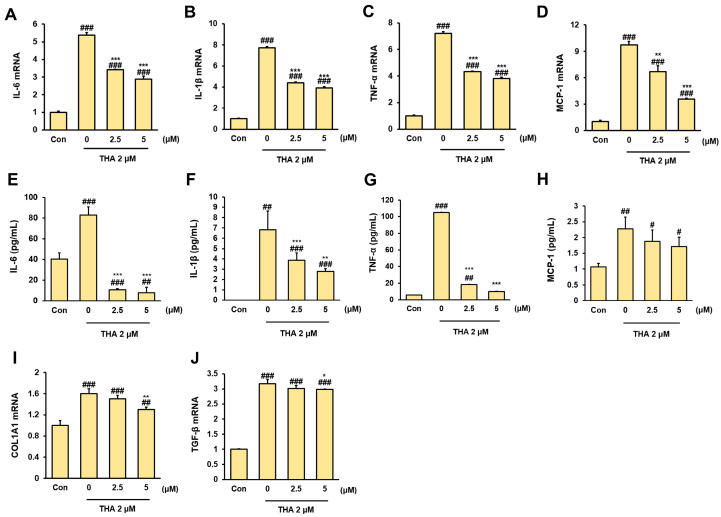
Anti-inflammatory effects of Rh2 treatment on ERS-induced HepG2 cells. mRNA expression of (**A**) IL-6, (**B**) IL-1β, (**C**) TNF-α, and (**D**) MCP-1 in HepG2 cells untreated (Con) or treated with 2 μM THA and (0 µM, 2.5 µM, 5 μM) Rh2 for 48 h. Quantitative analysis of CM (**E**) IL-6, (**F**) IL-1β, (**G**) TNF-α, and (**H**) MCP-1 from HepG2 cells was performed using a sandwich ELISA. (**I**,**J**) mRNA expression of fibrosis-related factors. The Con group was treated with DMSO, and the x-axis of the graph represents the Rh2 concentration. Values are shown as mean ± SD (*n* = 3). Con vs. 0, Con vs. 2.5, Con vs. 5, # *p* < 0.05, ## *p* < 0.01, ### *p* < 0.001. 0 vs. 2.5, 0 vs. 5, * *p* < 0.05, ** *p* < 0.01, *** *p* < 0.001.

When ERS occurs in hepatocytes, various inflammatory cytokines and chemokines are released. Therefore, we performed RT-PCR to monitor the mRNA expression of inflammatory factors in ERS-induced HepG2 cells. Upon ERS induction, the mRNA expression levels of IL-6, TNF-α, and IL-1β were significantly increased; however, these levels were markedly decreased following Rh2 treatment (Figure 4A–C, *p* < 0.001). Similar results were observed in the ELISA performed using CM derived from HepG2 cells. The levels of inflammatory cytokines were very high in the THA-treated group and significantly decreased when Rh2 was treated (Figure 4E–G). Interestingly, the mRNA expression of mcp-1, a chemokine important for recruiting macrophages and monocytes, was upregulated during ERS but significantly downregulated after Rh2 treatment (Figure 4D, *p* < 0.01, *p* < 0.001). This was similarly observed for MCP-1 levels in CM (Figure 4H). In addition, we confirmed that the mRNA expression of COL1A1 and TGF-b, factors affecting the liver fibrosis process, was upregulated by THA treatment but decreased by Rh2 treatment (Figure 4I,J). These results indicate that Rh2 can reduce the inflammatory response of hepatocytes and inhibit the secretion of inflammatory cytokines, thereby inhibiting macrophage recruitment and fibrosis progression.

### 3.3. Inhibition of THP-1 Cell Activation by CM Derived from ERS-Induced HepG2 Cells Treated with Rh2

**Figure 5:**
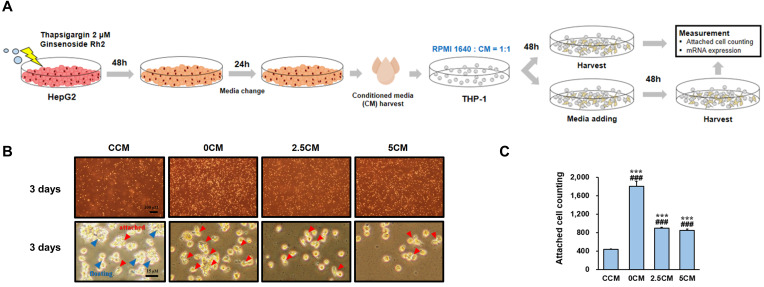
Inhibitory effect of conditioned medium (CM) from ERS-induced HepG2 cells treated with Rh2 on THP-1 cell differentiation. (**A**) The experiment where CM collected from HepG2 cells was treated to THP-1 cells is depicted. (**B**) Images of THP-1 cells cultured for 3 days under various medium conditions: Control CM from untreated HepG2 cells (CCM), CM from only ERS-induced HepG2 cells (0CM), and CM from ERS-induced HepG2 cells treated with 2.5 µM or 5 µM Rh2 (2.5CM and 5CM). Blue arrows indicate floating cells, and red arrows indicate attached cells. (**C**) Quantification of attached THP-1 cells. Values are shown as mean ± SD (*n* = 3). CCM vs. 0CM, CCM vs. 2.5CM, CCM vs. 5CM, ### *p* < 0.001. 0CM vs. 2.5CM, 0CM vs. 5CM, *** *p* < 0.001.

To determine whether factors released by ERS-induced HepG2 cells treated with Rh2 could affect THP-1 cells, we conducted an experiment in which CM from HepG2 cells was extracted and applied to THP-1 cells (Figure 5A). THP-1 cells were divided into four groups: a group treated with CM extracted from the Con group (CCM); a group treated with CM extracted from the 0 group (0CM); a group treated with CM extracted from the 2.5 group (2.5CM); a group treated with CM extracted from the 5 group (5CM). When observed under a microscope on the third day of culture, THP-1 cells in the CCM group were found to be floating in clumps with minimal attachment to the bottom, whereas cells in the 0CM group were attached to the bottom and differentiating into macrophages (Figure 5B). In addition, differentiation into macrophages was observed in the 2.5CM and 5CM groups; however, differentiation was reduced in these groups compared with the 0CM group. Similar results were obtained when the number of cells attached to the bottom was counted (Figure 5C). Therefore, Rh2 treatment inhibited the induction of THP-1 differentiation into macrophages by HepG2 cells.

### 3.4. Regulation of THP-1 Cell Polarization by CM Derived from ERS-Induced HepG2 Cells Treated with Rh2

**Figure 6:**
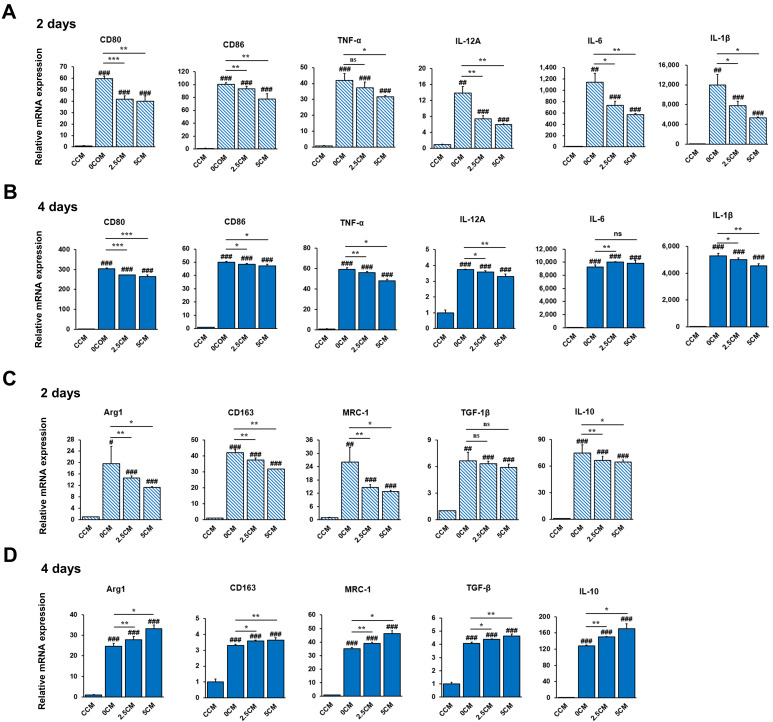
Effect of conditioned medium (CM) from ERS-induced HepG2 cells treated with Rh2 on the differentiation of THP-1 cells. mRNA expression of CD80, CD86, IL-1β, IL-12A, IL-6, and TNF-α in THP-1 cells treated with CM control CM from untreated HepG2 cells (CCM), CM from ERS-induced HepG2 cells (0CM), and CM from ERS-induced HepG2 cells treated with 2.5 µM or 5 µM Rh2 (2.5CM and 5CM) for (**A**) 2 days or (**B**) 4 days. mRNA expression of CD163, Arg1, TGF-1β, IL-10, and MRC1 in THP-1 cells treated with CM control CM from untreated HepG2 cells (CCM), CM from ERS-induced HepG2 cells (0CM), and CM from ERS-induced HepG2 cells treated with 2.5 µM or 5 µM Rh2 (2.5CM and 5CM) for (**C**) 2 days or (**D**) 4 days. Values are shown as mean ± SD (*n* = 3). CCM vs. 0CM, CCM vs. 2.5CM, CCM vs. 5CM, *# p* < 0.05, ## *p* < 0.01, ### *p* < 0.001. 0CM vs. 2.5CM, 0CM vs. 5CM, * *p* < 0.05, ** *p* < 0.01, *** *p* < 0.001.

To investigate the effect of CM from HepG2 cells on the differentiation phenotype, THP-1 cells cultured with CM for 2 or 4 days were harvested for RT-PCR analysis. The expression levels of M1 markers, including CD80, CD86, IL-1β, TNF-α, and IL-12α, were significantly decreased in the 2.5CM and 5CM groups on day 2 compared with the 0CM group on day 4 (Figure 6A,B). However, IL-6 was slightly increased in the 2.5CM group on day 4. The results demonstrated that CM from ERS-induced HepG2 cells treated with Rh2 inhibited the differentiation of liver-infiltrating macrophages into the M1 phenotype. The expression levels of M2 markers, such as MRC-1, CD163, Arg1, IL-10, and TGF-1β, were examined by RT-PCR. The levels of Arg1, TGF-1β, IL-10, and MRC-1 were significantly increased (*p* < 0.05, *p* < 0.01) when treated for 4 days compared with 2 days with CM (Figure 6C,D). However, the overall expression of CD163 was decreased in the 2.5CM and 5CM groups when treated for 4 days with CM, in contrast to 2 days. The results demonstrated that CM from ERS-induced HepG2 cells treated with Rh2 induced differentiation into the M2 phenotype.

## 4. Discussion

NAFLD is caused by a combination of factors, making it difficult to identify a therapeutic target [14]. Notably, although NAFL is characterized by excess lipid accumulation without inflammation, NASH involves hepatic inflammation, increasing the risk of progression to hepatocellular carcinoma [33]. Given the limited understanding of the mechanisms underlying the transition from NAFL to NASH, effective preventive and therapeutic strategies remain difficult to establish. Consequently, further research is essential to elucidate the pathogenesis of this transition.

Yao HR et al. [34] and Wang Q et al. [2] demonstrated that free fatty acids and ERS accelerate fatty liver formation and trigger inflammatory responses, which significantly contribute to the progression of NASH. Therefore, to establish a cell model of NAFLD, we first treated HepG2 cells with PA, EtOH [35], and THA (an ERS inducer) to identify substances that induce lipid accumulation. Among them, THA effectively induced ERS in HepG2 cells. Furthermore, THA treatment upregulated the inflammatory markers IL-1β, IL-6, and TNF-α, as well as key genes involved in the lipid biosynthesis pathway, including SREBP1 and FAS, leading to increased lipid accumulation.

In our search for a natural compound that could inhibit and treat the ERS-accelerated progression from NAFL to NASH, we focused on ginsenosides, known for their anti-inflammatory effects [27,28]. Notably, previous research demonstrated that Rh2 could reduce ERS in lung cancer cells [36]. Similarly, in our study, Rh2 treatment reduced apoptosis, downregulated ERS-related factors, and suppressed pro-inflammatory cytokines in THA-treated HepG2 cells. These findings suggest that Rh2 treatment may mitigate hepatocyte damage associated with liver inflammation during the early transition from NAFL to NASH. Although recent research has demonstrated that Rh2 can inhibit adipocyte differentiation [10], its effect on reducing lipid accumulation in hepatocytes remains unexplored. In our study, morphological and quantitative assessments via Oil Red O staining and triglyceride assay, as well as evaluation of SREBF1/FAS mRNA expression, demonstrated that Rh2 significantly inhibited the expression of lipid synthesis-related genes and mitigated lipid accumulation in HepG2 cells.

Interestingly, we also observed that MCP-1 expression, a key factor in macrophage recruitment and infiltration [37], was more than 10-fold higher in the CM of THA-treated HepG2 cells than in controls and was significantly reduced by Rh2 treatment. These findings suggest that ERS may facilitate macrophage recruitment in hepatocytes, whereas Rh2 treatment may counteract this effect. A critical aspect of macrophage function in NASH development is polarization toward either the M1 or M2 phenotype. Recent studies have shown that macrophage infiltration and activation, particularly polarization toward the M1 phenotype, increase during the transition from NAFL to NASH [38]. M1 macrophages are pro-inflammatory, releasing cytokines such as TNF-α, IL-1β, and IL-6 [39], which exacerbate liver damage and promote the progression to NASH. In contrast, M2 macrophages are associated with anti-inflammatory functions, secreting cytokines such as TGF-1β and IL-10, which help to resolve inflammation and facilitate tissue repair [40,41]. During the transition from NAFL to NASH [42], an imbalance occurs with increased M1 polarization, leading to sustained chronic inflammation and enhanced fibrogenesis. Therefore, modulation of the M1/M2 macrophage balance has been identified as a potential therapeutic strategy to mitigate NASH progression. In our study, the treatment of THP-1 cells with CM significantly reduced the expression of M1 phenotype markers in the 2.5CM and 5CM groups compared with the 0CM group. This reduction was more pronounced after 4 days of treatment than after 2 days. In contrast, the expression of M2 phenotype markers increased in the 2.5CM and 5CM groups, with a more pronounced effect after 4 days than after 2 days. These results suggest that Rh2 regulates ERS-induced HepG2 cells, promoting M2 differentiation while suppressing M1 differentiation. These findings are partially consistent with previous findings showing that Rh2, in combination with MSC-derived exosomes, suppressed M1 polarization and promoted M2 differentiation in inflammatory disease models [43].

Collectively, our findings suggest that Rh2 treatment of HepG2 cells could inhibit lipid synthesis and inflammatory responses, thus protecting hepatocytes and potentially preventing NAFLD progression by modulating the interaction between HepG2 cells and THP-1 monocytes. Although a limitation of this study is the inability to determine the precise ratio of M1 to M2 macrophages in CM-treated THP-1 cells, our results suggest that Rh2 treatment could promote the expression of M2 macrophage-related factors, which are associated with anti-inflammatory and tissue repair functions, rather than M1 macrophage-related pro-inflammatory factors that exacerbate inflammation. Therefore, Rh2 may have therapeutic potential in mitigating NAFLD progression.

Moreover, our study emphasizes the importance of hepatocyte-derived inflammatory signals in macrophage polarization. The marked reduction in MCP-1 and other pro-inflammatory cytokines following Rh2 treatment indicates that Rh2 may suppress paracrine signaling that recruits and activates monocytes. Given the central role of chronic inflammation in NAFLD progression, the ability of Rh2 to modulate immune cell responses indirectly through hepatocyte signaling provides valuable insight into its therapeutic potential.

Although our findings highlight the promising therapeutic effects of Rh2 on NAFLD in vitro, its pharmacokinetic limitations must be acknowledged. According to pharmacokinetic studies, many ginsenosides, including Rh2, exhibit very low oral bioavailability (less than 5%) due to poor gastrointestinal absorption [44,45]. Despite this limitation, Rh2 has demonstrated significant pharmacological activity in various in vitro models, and in vivo studies have reported promising outcomes, such as reduced tumor incidence in mice following oral administration [46,47]. These findings motivated our investigation into Rh2, and additional studies focusing on its oral absorption and systemic bioavailability could further support its development as a therapeutic agent and its potential translation into clinical use.

While our findings are based on an in vitro model, they suggest that Rh2 could be effective in mitigating early inflammatory responses and preventing the fibrotic remodeling observed in NASH. Future studies employing in vivo NAFLD models are warranted to validate these findings and assess the long-term metabolic and histological benefits of Rh2 administration.

## 5. Conclusions

This study demonstrates that the ginsenoside Rh2 alleviates hepatic lipotoxicity and inflammation by attenuating ERS and modulating hepatocyte–macrophage interactions in an in vitro model of NAFLD. Rh2 suppressed THA-induced lipid accumulation, ERS markers, and inflammatory cytokine expression in HepG2 cells, while the conditioned medium from Rh2-treated hepatocytes inhibited THP-1 differentiation into M1-type macrophages and promoted M2 polarization. These findings suggest that Rh2 not only protects hepatocytes from ER stress-induced damage, but also indirectly regulates immune responses in the liver microenvironment. Therefore, Rh2 holds therapeutic potential as a preventive or adjunctive agent in the early management of NAFLD, and possibly its progression to NASH.

## Figures and Tables

**Table 1:** Primer sequences used in RT-PCR.

Gene		Primer Sequences	Accession Number
*β-actin*	Forward (5′-3′)	GATTCCTATGTGGGCGACGA	NM_001101.5
	Reverse (5′-3′)	TCTCCATGTCGTCCCAGTTG	
*IL-1β*	Forward (5′-3′)	CTCTGTCATTCGCTCCCACA	XM_054341810.1
	Reverse (5′-3′)	ACACTGCTACTTCTTGCCCC	
*IL-6*	Forward (5′-3′)	AGTGAGGAACAAGCCAGAGC	NM_000600.5
	Reverse (5′-3′)	ATTTGTGGTTGGGTCAGGGG	
*TNF-α*	Forward (5′-3′)	GTCCTCTTCAAGGGCCAAGG	NM_000594.4
	Reverse (5′-3′)	GGCTCTTGATGGCAGAGAGG	
*FAS*	Forward (5′-3′)	GGCCCACAAGAGCTACATCA	XM_054315477.1
	Reverse (5′-3′)	GGAGCGAGAAGTCAACACGA	
*SREBF1*	Forward (5′-3′)	TGACCGACATCGAAGGTGAA	NM_001005291.3
	Reverse (5′-3′)	AAAGTGCAATCCATGGCTCC	
*FOXA3*	Forward (5′-3′)	TCTTGGGGCCTGATCCTTCT	NM_004497.3
	Reverse (5′-3′)	GGATCAACACCATGCCCACT	
*BiP*	Forward (5′-3′)	TGAAAGAAACCGCTGAGGCT	NM_005347.5
	Reverse (5′-3′)	TCTTTGGTTGCTTGGCGTTG	
*MCP-1*	Forward (5′-3′)	GCAGTAAGTGTCCCAAAGAAGC	NM_002982.4
	Reverse (5′-3′)	TGGGTTTGCTTGTCCAGGTG	
*COL1A1*	Forward (5′-3′)	GCTGGTGCTCGTGGAAAT	NM_000088.4
	Reverse (5′-3′)	ACCCTTAGCACCAACAGC	
*CD86*	Forward (5′-3′)	CTTCCTGCTCTCTGGTGCTG	NM_176892.2
	Reverse (5′-3′)	GCTCACTCAGGCTTTGGTTC	
*CD80*	Forward (5′-3′)	CCACAACCTTTGGAGACCCA	NM_005191.4
	Reverse (5′-3′)	AGGCAGGGCTGATGACAATC	
*IL-12A*	Forward (5′-3′)	GCTCCAGAAGGCCAGACAAA	NM_000882.4
	Reverse (5′-3′)	TAAACAGGCCTCCACTGTGC	
*CD163*	Forward (5′-3′)	GGACCCACTTCCTGTTCTGG	XM_054373862.1
	Reverse (5′-3′)	TGACACACCACCTGAGCATC	
*ARG1*	Forward (5′-3′)	GGGTTGACTGACTGGAGAGC	NM_000045.4
	Reverse (5′-3′)	CGTGGCTGTCCCTTTGAGAA	
*MRC1*	Forward (5′-3′)	GGGACGTGGCTGTGGATAAA	NM_002438.4
	Reverse (5′-3′)	TCCAAAACCCAGAAGACGCA	
*IL-10*	Forward (5′-3′)	CGAGATGCCTTCAGCAGAGT	NM_000572.3
	Reverse (5′-3′)	GGCAACCCAGGTAACCCTTA	

## Data Availability

The data are contained within the article.
